# Maternal History of Weight Loss and Prospective Gestational Weight Gain

**DOI:** 10.1001/jamanetworkopen.2026.7931

**Published:** 2026-04-20

**Authors:** Meghan E. Muse, Diane Gilbert-Diamond, Juliette Madan, Janet L. Peacock, Margaret R. Karagas, Caitlin G. Howe

**Affiliations:** 1 Department of Epidemiology, Geisel School of Medicine at Dartmouth, Lebanon, New Hampshire; 2 Department of Psychiatry, Dartmouth Health, Dartmouth Hitchcock Medical Center, Lebanon, New Hampshire; 3 Department of Pediatrics, Dartmouth Health, Dartmouth Hitchcock Medical Center, Lebanon, New Hampshire

## Abstract

**Importance:**

Higher gestational weight gain is associated with adverse outcomes, including macrosomia. Weight cycling (cycles of weight loss followed by weight regain) is associated with increased risk of future weight gain, but little is known about how history of weight cycling is associated with pregnancy weight gain.

**Objective:**

To assess whether an association exists between weight cycling history and gestational weight gain in a US pregnancy cohort.

**Design, Setting, and Participants:**

This cohort study used data collected from participants recruited from prenatal clinics in New Hampshire for The New Hampshire Birth Cohort Study, an ongoing prospective cohort study initiated in 2009. Eligibility for the study included being between 18 and 45 years of age and experiencing a singleton pregnancy. The present analysis took place between April 2024 and September 2025 and reflects participants enrolled prior to June 2021.

**Exposure:**

History of weight cycling, defined as the number of times an individual self-reported losing 20 pounds (9.1 kg) or more in adulthood (excluding after pregnancy).

**Main Outcomes and Measures:**

The primary outcome was total gestational weight gain.

**Results:**

Of 2628 enrolled participants, 1188 with complete exposure, outcome, and covariate data were included in the analytic sample. Among them, the mean (SD) age was 31.5 (4.7) years, and the mean (SD) prepregnancy body mass index (BMI; calculated as weight in kilograms divided by height in meters squared) was 25.6 (5.6). Approximately half the participants (n = 561 [47.2%]) reported any history of weight cycling, and the mean (SD) gestational weight gain was 16.9 (6.5) kg. In linear models adjusting for highest lifetime BMI, age, educational attainment, and smoking history, individuals reporting 1, 2, and 3 or more weight cycles gained a mean of 1.7 (95% CI, 0.8-2.6) kg, 3.2 (95% CI, 2.0-4.5) kg, and 6.2 (95% CI, 4.7-7.7) kg more, respectively, over their entire pregnancy than individuals who reported no history of weight cycling (*P* < .001 for trend).

**Conclusions and Relevance:**

This cohort study of pregnant individuals found that a history of weight cycling was positively associated with gestational weight gain, independent of BMI. These findings may have important clinical implications for identifying individuals at high risk of excessive gestational weight gain. Additional research is needed to assess potential implications for maternal and child health.

## Key Points

**Question:**

Is there an association between maternal history of weight cycling and gestational weight gain?

**Findings:**

In this cohort study of 1188 pregnant individuals, history of weight cycling was associated with higher gestational weight gain in a dose-dependent manner. Individuals with 3 or more weight cycles gained a mean of 6.2 kg more during pregnancy than those with no history of weight cycling.

**Meaning:**

In this study, history of weight cycling was associated with higher weight gain during pregnancy, which is known to increase risk of adverse infant and maternal outcomes, such as higher infant birth weight and postpartum weight retention.

## Teaser

This cohort study investigates whether an association exists between a history of weight loss and regain cycles and gestational weight gain among pregnant individuals.

## Introduction

Both obesity and gestational weight gain in excess of 2009 Institute of Medicine (IOM) guidelines^1^ have been associated with adverse infant and maternal outcomes, such as higher infant birth weight (including macrosomia and large-for-gestational-age infants) and higher postpartum weight retention.^2,3,4,5,6,7,8,9^ One factor associated with obesity and future weight gain is a history of weight cycling.

Weight cycling, often referred to as yo-yo dieting, is the cyclical process of weight loss followed by weight regain. Prior studies have estimated that 20% to 55% of women have history of weight cycling,^10^ and weight cycling practices have been associated with higher weight gain, higher body mass index (BMI; calculated as weight in kilograms divided by height in meters squared), and higher rates of obesity later in life.^11,12,13^ Prior work in the Nurses’ Health Study II found that women who reported severe weight cycling, defined as reporting losing at least 20 pounds (9.1 kg) 3 or more times, had a mean BMI approximately 9 units higher than women who reported no history of weight cycling.^14^ Weight cycling is also associated with multiple adverse health conditions, including diabetes, hypertension, and cardiometabolic events.^15,16,17,18^ In animal models, induced weight cycling behavior also contributed to metabolic dysregulation.^19,20,21,22^

Currently, it is unknown whether history of weight cycling is associated with weight status and subsequent weight gain in the context of pregnancy. Here, we assessed the association between reported history of weight cycling in adulthood and prospective gestational weight gain among a sample of pregnant individuals enrolled in the New Hampshire Birth Cohort Study.^23^ Given the established associations between history of weight cycling and future weight gain, we hypothesized that individuals who report a history of weight cycling experience greater weight gain during pregnancy and are at greater risk of gestational weight gain in excess of IOM guidelines.

## Methods

### Study Population

Starting in January 2009, pregnant individuals at approximately 24 through 28 weeks’ gestation were recruited into the New Hampshire Birth Cohort Study, a prospective cohort study, from prenatal clinics in New Hampshire, as previously described.^23^ Inclusion criteria included being between 18 and 45 years of age, having a singleton pregnancy, and having no plans to move during pregnancy. The present analysis took place between April 2024 and September 2025 and reflects participants who were both enrolled and had follow-up through delivery prior to June 2021. All study protocols were approved by the institutional review board at Dartmouth College, and written informed consent was obtained from all participants at enrollment. This report followed the Strengthening the Reporting of Observational Studies in Epidemiology (STROBE) reporting guideline for cohort studies.

### Exposure and Outcome Assessment

At enrollment, participants completed a study questionnaire that collected prepregnancy weight, highest weight since turning 18 years of age (excluding during pregnancy), and total times that they had lost 20 pounds (9.1 kg) or more since turning 18 years of age (excluding postpartum weight loss). History of weight cycling was derived from participant responses to the question, “Since you were 18 years old, how many times have you lost more than 20 pounds (except after pregnancy)?” Responses to this question were categorized as 0, 1, 2, or 3 or more times.

The primary outcome was total gestational weight gain. Participant height was abstracted from medical records and used to calculate prepregnancy BMI and highest BMI in adulthood. Pregnancy weights were abstracted from prenatal medical records, and total gestational weight gain was calculated as the difference between the final weight collected up to 7 days prior to delivery (mean, 3 days prior to delivery) and self-reported prepregnancy weight. Using the participant’s prepregnancy BMI, continuous gestational weight gain was categorized as inadequate, adequate, or excessive according to the IOM guidelines for weight gain based on prepregnancy BMI.^1^

### Covariate Assessment

Participants self-reported race and ethnicity, their highest attained level of education, and smoking status (current, former, or never) via questionnaire at enrollment. Diagnosis of gestational diabetes or gestational hypertension during the current pregnancy as well as diagnosis of diabetes or hypertension prior to the current pregnancy were abstracted from prenatal medical records. Race and ethnicity were assessed to more fully characterize the cohort. Participants selected from race categories of American Indian or Alaska Native, Asian or Pacific Islander, Black or African American, White or Caucasian, and other and from ethnicity categories of Hispanic or non-Hispanic, as indicated by the National Institutes of Health guidelines.

### Statistical Analysis

All statistical analyses were conducted in R, version 4.5.1 (R Project for Statistical Computing). Covariate-adjusted linear regression was used to model the association between history of weight cycling (variable, categorical) and gestational weight gain (outcome, continuous). Additionally, covariate-adjusted multinomial logistic regression was conducted using the nnet package (version 7.3-20) in R to model the association between history of weight cycling (variable, categorical) and inadequate vs adequate or excessive vs adequate gestational weight gain per IOM guidelines for weight gain based on prepregnancy BMI categories.^1^ Binned residuals were plotted against predicted values using the arm package (version 1.14-4) in R to assess model assumptions.

Model covariates were selected using a directed acyclic graph (eFigure 1 in Supplement 1). All models were adjusted for the participant’s highest attained BMI in adulthood, educational attainment, smoking history (ever, never, or unreported), and age at enrollment. To test for a linear trend in the association between history of weight cycling and gestational weight gain, all models were repeated treating history of weight cycling as an ordinal variable. Restricted cubic splines with 4 knots were implemented using the rms package (version 8.1-0) in R to assess possible nonlinear associations with BMI and age via a likelihood ratio test comparing a model with a spline to a nested model with only a linear term for BMI or age, respectively. In subsequent models, BMI was modeled using a restricted cubic spline due to its identified nonlinear relationship with weight gain, and age was modeled linearly.

A series of sensitivity analyses was conducted, including (1) excluding participants with a self-reported prepregnancy BMI less than 18.5 (n = 24), (2) excluding participants with a history of diabetes prior to the pregnancy (n = 45), (3) excluding participants with a history of hypertension prior to the pregnancy (n = 218), (4) adjusting for prepregnancy BMI instead of the highest BMI in adulthood, and (5) without adjusting for BMI. In an exploratory analysis, we tested for a possible interaction between weight cycling history and prepregnancy weight status. These models were restricted to participants with a prepregnancy BMI higher than 18.5, and weight status was classified as normal weight (BMI >18.5 and <25.0) or overweight or obesity (BMI ≥25.0). Associations were modeled as in the aforementioned primary analysis, adjusting for prepregnancy weight status, educational attainment, smoking history, and age at enrollment. A likelihood ratio test was conducted comparing a model fitting an interaction term between weight cycling history and weight status to a nested model with no interaction term. A 2-sided *P* < .05 was considered statistically significant.

## Results

### Participant Demographics

**Figure 1.**
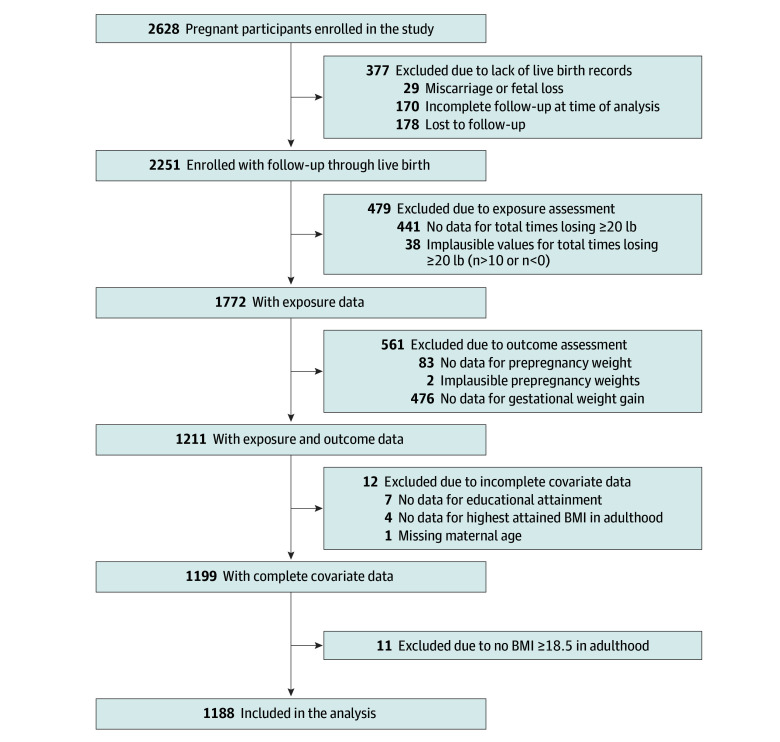
Participant Flowchart BMI indicates body mass index (calculated as weight in kilograms divided by height in meters squared).

From a total of 2628 enrolled participants in the New Hampshire Birth Cohort at the time that the statistical analysis was initiated, 1188 had complete exposure, outcome, and covariate data and were included in the final analyses (Figure 1). Among them, the mean (SD) age was 31.5 (4.7) years, and 1148 (96.8%) self-reported being White, with 23 (1.9%) self-reporting being mixed race, and the other sample sizes being too small to report; 1152 (97.7%) self-reported being non-Hispanic (Table 1). The mean (SD) prepregnancy BMI was 25.6 (5.6). Fewer than half of participants (n = 561 [47.2%]) reported any history of weight cycling, with 110 participants (9.3%) reporting 3 or more instances of losing 20 pounds (9.1 kg) or more in adulthood (excluding pregnancy). Participants with a history of weight cycling had a higher prepregnancy BMI and highest reported BMI in adulthood and were also more likely to report a history of ever smoking. Parity and gestational duration of the current pregnancy did not differ by history of weight cycling category. Overall, participants gained a mean (SD) of 16.9 (6.5) kg during pregnancy (Table 1 and eFigure 2 in Supplement 1). The analytic subset did not differ from the overall study population with the exception of having a lower proportion of college graduates, a higher proportion of smokers, a higher gestational age at delivery, and fewer preterm deliveries (eTable 1 in Supplement 1). No associations were observed between the exposure and missingness of the outcome or between the outcome and missingness of the exposure (eTable 2 in Supplement 1).

**Table 1.** Participant Demographics and Pregnancy Characteristics Stratified by Total Reported Weight Cycles^a^

Characteristic	Overall	Total No. of weight cycles				*P* value^b^
		0	1	2	≥3	
Sample size	1188 (100)	627 (52.8)	298 (25.1)	153 (12.9)	110 (9.3)	NA
Age, mean (SD), y	31.49 (4.67)	31.50 (4.62)	31.09 (4.78)	31.53 (4.61)	32.46 (4.72)	.08
Educational attainment						
≤High school graduate	135 (11.4)	61 (9.7)	39 (13.1)	20 (13.1)	15 (13.6)	.01
Some college	194 (16.3)	88 (14.0)	46 (15.4)	34 (22.2)	26 (23.6)	
≥College graduate	859 (72.3)	478 (76.2)	213 (71.5)	99 (64.7)	69 (62.7)	
Race^c^						
American Indian or Alaska Native	NR	NR	NR	0	0	.55
Asian	NR	NR	0	NR	0	
Black	NR	NR	NR	0	0	
White	1148 (96.8)	600 (96.0)	289 (97.0)	151 (98.7)	108 (98.2)	
Mixed race	23 (1.9)	15 (2.4)	NR	0	NR	
Ethnicity^d^						
Hispanic	27 (2.3)	15 (2.4)	NR	NR	NR	.96
Non-Hispanic	1152 (97.7)	606 (97.6)	290 (98.0)	150 (98.0)	106 (97.2)	
Parity^e^						
1	518 (43.7)	256 (40.9)	148 (50.0)	66 (43.1)	48 (43.6)	.02
2	446 (37.6)	254 (40.6)	105 (35.5)	54 (35.3)	33 (30.0)	
≥3	221 (18.6)	116 (18.5)	43 (14.5)	33 (21.6)	29 (26.4)	
Smoking status^f^						
Never	963 (89.0)	532 (92.4)	238 (87.8)	113 (83.7)	80 (80.0)	<.001
Ever	119 (11.0)	44 (7.6)	33 (12.2)	22 (16.3)	20 (20.0)	
Highest adult BMI, mean (SD)^g^	27.79 (6.48)	24.86 (4.08)	29.01 (6.35)	31.78 (6.65)	35.62 (7.26)	<.001
Prepregnancy BMI, mean (SD)^g^	25.58 (5.60)	23.63 (3.91)	26.23 (6.18)	28.38 (5.96)	31.02 (5.99)	
<18.5	24 (2.0)	17 (2.7)	5 (1.7)	2 (1.3)	0	<.001
18.5 to <25.0	671 (56.5)	457 (72.9)	152 (51.0)	44 (28.8)	18 (16.4)	
25.0-30.0	286 (24.1)	106 (16.9)	83 (27.9)	59 (38.6)	38 (34.5)	
>30	207 (17.4)	47 (7.5)	58 (19.5)	48 (31.4)	54 (49.1)	
Total weeks’ gestation, mean (SD)	39.15 (1.50)	39.21 (1.52)	39.16 (1.51)	39.14 (1.43)	38.81 (1.47)	.09
Total weight gain, mean (SD), kg	16.9 (6.5)	16.0 (5.5)	17.2 (6.2)	18.0 (7.2)	20.0 (9.3)	<.001
Weight gain defined by IOM guidelines						
Inadequate	109 (9.2)	68 (10.8)	26 (8.7)	6 (3.9)	9 (8.2)	<.001
Adequate	312 (26.3)	217 (34.6)	63 (21.1)	22 (14.4)	10 (9.1)	
Excessive	767 (64.6)	342 (54.5)	209 (70.1)	125 (81.7)	91 (82.7)	

Abbreviations: BMI, body mass index; IOM, Institute of Medicine; NA; not applicable; NR, not reported.

^a^ Data given as No. (%) except where noted.

^b^ *P* values obtained in analysis of variance for continuous variables and a χ^2^ test for categorical variables.

^c^ Missing for 2 participants; data not reported when there were only a few participants, to maintain anonymity.

^d^ Missing for 9 participants; data not reported when there were only a few participants, to maintain anonymity.

^e^ Missing for 3 participants.

^f^ Missing for 106 participants.

^g^ BMI calculated as weight in kilograms divided by height in meters squared.

### Association of Weight Cycling History With Gestational Weight Gain

**Figure 2.**
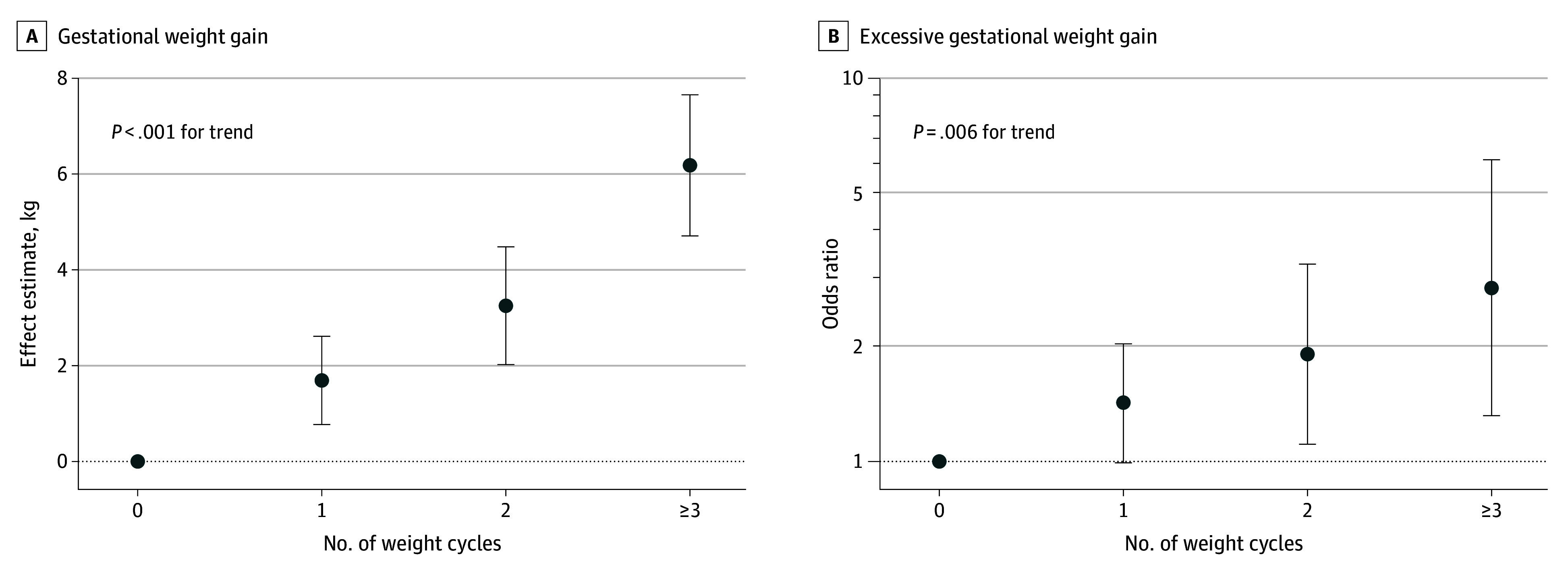
Dot Plots Depicting Covariate-Adjusted Associations Between History of Weight Cycling and Total Gestational Weight Gain, and Odds of Excessive Gestational Weight Gain (Relative to Adequate Gestational Weight Gain), Per Institute of Medicine Guidelines Models are adjusted for self-reported highest attained body mass index in adulthood (excluding pregnancy; continuous), educational attainment (categorical), and maternal age (continuous). *P* for trend reflects the *P* value for the linear term when history of weight cycling is treated as an ordinal variable. Error bars indicate 95% CI.

Individuals reporting 1, 2, and 3 or more weight cycles gained a mean of 1.7 (95% CI, 0.8-2.6) kg, 3.2 (95% CI, 2.0-4.5) kg, and 6.2 (95% CI, 4.7-7.7) kg more, respectively, over their entire pregnancy than individuals who reported no history of weight cycling (*P* < .001 for trend) (Figure 2) after adjusting for highest reported BMI in adulthood, educational attainment, smoking history, and maternal age (Table 2). Effect estimates were similar and remained statistically significant in sensitivity analyses (eTable 3 in Supplement 1).

**Table 2.** Associations Between History of Weight Cycling and Gestational Weight Gain

Weight cycle, No.^b^	Crude association		Adjusted association^a^	
	Effect estimate (95% CI)	*P* value	Effect estimate (95% CI)	*P* value
**Total gestational weight gain (kg)^c^**				
0	[Reference]	[Reference]	[Reference]	[Reference]
1	1.2 (0.3-2.1)	<.001	1.7 (0.8-2.6)	<.001
2	2.0 (0.9-3.1)	<.001	3.2 (2.0-4.5)	<.001
≥3	4.0 (2.7-5.3)	<.001	6.2 (4.7-7.7)	<.001
**ORs for inadequate (relative to adequate) gestational weight gain^d^**				
0	[Reference]	[Reference]	[Reference]	[Reference]
1	1.32 (0.77-2.24)	.31	1.05 (0.59-1.88)	.87
2	0.87 (0.34-2.23)	.77	0.48 (0.17-1.34)	.16
≥3	2.87 (1.12-7.36)	.03	1.20 (0.40-3.60)	.75
**ORs for excessive (relative to adequate) gestational weight gain^d^**				
0	[Reference]	[Reference]	[Reference]	[Reference]
1	2.10 (1.51-2.93)	<.001	1.41 (0.99-2.02)	.06
2	3.61 (2.22-5.85)	<.001	1.90 (1.11-3.26)	.02
≥3	5.77 (2.94-11.34)	<.001	2.82 (1.31-6.08)	.008

Abbreviations: IOM, Institute of Medicine (2009); ORs, odds ratios.

^a^ Models are adjusted for self-reported highest attained body mass index in adulthood (excluding pregnancy; continuous; modeled with restricted cubic splines with 4 knots), educational attainment (categorical), smoking history (categorical), and maternal age (continuous).

^b^ Weight cycles are modeled categorically and are defined as the total number of times a mother reported losing 20 pounds (9.1 kg) or more, excluding after a pregnancy.

^c^ Modeled using linear regression.

^d^ Per IOM weight guidelines. Modeled using multinomial logistic regression; effect estimates are exponentiated to reflect ORs.

To assess the possible contribution of differences in underreporting of prepregnancy BMI between weight cycling classifications, we also assessed gestational weight gain using weights abstracted from medical records during the first trimester of pregnancy. Participants with available first trimester weights tended to be more highly educated and were more likely to be primiparous than those that did not (eTable 4 in Supplement 1). The mean (SD) gestational age of the earliest first trimester weight was 8.7 (1.6) weeks, and participants who reported a history of weight cycling had greater differences between their medical record abstracted first trimester weight and self-reported prepregnancy weight (eFigure 3 in Supplement 1). The strength of association between history of weight cycling and gestational weight gain was attenuated when calculating gestational weight gain using first trimester weights abstracted from medical records instead of self-reported weight (eFigure 3 in Supplement 1). There was no evidence of effect modification by prepregnancy weight status on the association between history of weight cycling and gestational weight gain (eFigure 4 and eTable 5 in Supplement 1).

### Association of Weight Cycling History With Inadequate vs Adequate Gestational Weight Gain, Per IOM Guidelines

In unadjusted models, individuals reporting 3 or more weight cycles had 2.87 (95% CI, 1.12-7.36) times the odds of inadequate gestational weight gain per IOM guidelines than individuals who did not report a history of weight cycling. However, this association was no longer statistically significant after adjusting for the participant’s highest BMI in adulthood, educational attainment, smoking history, and age (odds ratio, 1.20 [95% CI, 0.40-3.60]) (Table 2).

### Association of Weight Cycling History With Excessive vs Adequate Gestational Weight Gain, Per IOM Guidelines

There was a statistically significant positive linear trend for the association between reported history of weight cycling and excessive gestational weight gain (*P* = .006 for trend) (Figure 2). Among individuals reporting 1, 2, and 3 or more weight cycles, the adjusted odds of gaining weight in excess of IOM guidelines were 1.41 (95% CI, 0.99-2.02), 1.90 (95% CI, 1.11-3.26), and 2.8 (95% CI, 1.31-6.08) times that of individuals who reported no history of weight cycling, respectively (Table 2). There was no clear suggestion that model assumptions were violated; 787 projected categorizations (66.2%) agreed with the observed, indicating moderate accuracy (eFigure 5 and eTable 6 in Supplement 1). These findings were robust in sensitivity analyses, and the odds ratios were highest in models that did not account for BMI (eTable 7 in Supplement 1). Additionally, associations between history of weight cycling and excessive gestational weight gain were consistent when calculating gestational weight gain using first trimester weights abstracted from medical records instead of self-reported weights (eFigure 3 in Supplement 1). There was no evidence of effect modification by prepregnancy weight status on the association between history of weight cycling and gaining weight in excess of IOM guidelines (eFigure 4 and eTable 5 in Supplement 1).

## Discussion

In this prospective US pregnancy cohort, we identified a positive association between self-reported history of weight cycling, as defined by the number of times an individual reported losing 20 pounds (9.1 kg) or more in adulthood (excluding after a pregnancy), and both prospective gestational weight gain and the odds of gaining weight in excesses of IOM guidelines. These findings were robust to covariate adjustment and multiple sensitivity analyses.

Consistent with prior literature assessing BMI in nonpregnant populations,^11,12,13,14^ individuals in our study who reported a history of weight cycling had a higher prepregnancy BMI, as assessed by self-report of prepregnancy weight. Our finding that history of weight cycling was associated with higher gestational weight gain aligns with prior findings in nonpregnant populations that identified associations between weight cycling history and future weight gain.^12,13,24^ In addition to observing higher overall gestational weight gain in individuals who reported a history of weight cycling, we observed that these individuals were also more likely to experience weight gain during their pregnancy that was in excess of IOM guidelines. These findings could have important clinical implications, as they may help to identify populations at higher risk for excessive gestational weight gain that may benefit from tailored guidelines or interventions. More research is needed to evaluate these associations in additional populations and to compare different measures of weight cycling history. However, there may be benefit to health care professionals asking obstetric patients about weight cycling history in addition to prepregnancy weight during their initial prenatal visits, following existing recommendations for minimizing stigma.^25^ Additionally, given prior literature that found associations between weight cycling in nonpregnant populations and diabetes and hypertension,^15,16,17,18^ as well as metabolic dysregulation in animal models,^19,20,21,22^ it is possible that individuals with a history of weight cycling may be at higher risk of pregnancy complications associated with excessive gestational weight gain, although additional research is needed in this area. As pregnancy complications such as gestational diabetes and gestational hypertension have been associated with infant anthropometry at birth as well as body composition later in life,^26,27,28^ there may also be important implications for the future metabolic health of the infant.

While this is the first study to our knowledge assessing history of weight cycling with gestational weight gain, prior work has assessed changes in gestational weight gain with other changes to weight prior to pregnancy. For example, a recent study demonstrated that women who had been prescribed glucagon-like peptide-1 receptor agonists, a cause of marked weight loss, prior to pregnancy had increased gestational weight gain and an increased risk of excessive gestational weight gain compared with matched controls on a similar scale to that observed in our population.^29^ A few studies have also assessed gestational weight gain in women who have previously undergone bariatric surgery; however, findings have been mixed, possibly due to population differences in prepregnancy BMI of the control group.^30,31,32^ Taken together, these findings suggest that marked weight loss prior to pregnancy may impact gestational weight gain, which may have important implications for both maternal and child health.

### Strengths and Limitations

This study was strengthened by its large sample size, prospective design, robust covariate adjustment, and use of clinical measurements of gestational weight abstracted from medical records. Additionally, this study assessed gestational weight gain using 2 distinct measures of prepregnancy weight: self-report and first trimester weight from medical records. As these weights are what are commonly available to clinicians based on the timing of initial prenatal visits, these findings are directly relevant to clinical applications of gestational weight gain guidelines.

This study has limitations. The most notable was the use of self-report to assess prepregnancy weight, highest attained weight during adulthood, and total number of weight cycles. While maternal weights were abstracted from medical records during pregnancy, total gestational weight gain was calculated using self-reported prepregnancy weight due to variation in the collection of the first weight during pregnancy. Given the previously established tendency for pregnant individuals to underreport their prepregnancy weight,^33^ it is likely that calculated gestational weight gain was generally overestimated for participants. While prior research did not find an association between prepregnancy weight status and misreporting of prepregnancy weight,^34^ the association between history of weight cycling and misreporting of weight has not yet been assessed. When using the first weight abstracted from medical records from the first trimester of pregnancy as a proxy for prepregnancy weight, we observed greater evidence of possible underreporting of prepregnancy weight among participants with a history of weight cycling. However, as most of the first measurements of weight during pregnancy from medical records came from the second half of the first trimester, we cannot assess whether these discrepancies were truly a product of underreporting or if they were a consequence of differences in early pregnancy weight gain among individuals with a history of weight cycling. Importantly, our findings were consistent although attenuated when assessing gestational weight gain using only early pregnancy weights abstracted from medical records rather than self-reported prepregnancy weight. Future work is needed to assess if differential self-reporting bias occurs among pregnant individuals by weight history, including history of weight cycling.

While no evidence of effect modification was observed by prepregnancy weight status, this analysis may have been underpowered due to a small sample size among individuals with a prepregnancy BMI between 18.5 and 25.0 and with the highest exposure to weight cycling. Thus, additional work is needed to assess whether the association between history of weight cycling and gestational weight gain is modified by prepregnancy BMI.

Due to our approach used to assess weight cycling, we were unable to differentiate between intentional vs unintentional weight loss. We were also unable to assess the cause of weight loss (eg, lifestyle vs weight loss medications) and did not have information to determine whether a participant experienced weight loss due to an eating disorder. Additional work is therefore needed to identify the causes of weight cycling and weight loss history in pregnant individuals to improve understanding of how history of weight cycling is associated with future weight gain. Future work is also needed to assess the association between weight cycling history and pregnancy complications that have previously been linked to gestational weight gain to better understand whether excess gestational weight gain is consistently associated with adverse outcomes in individuals with a history of weight cycling.

As the New Hampshire Birth Cohort recruits from prenatal clinics in New Hampshire, the present analysis focused on a predominantly rural population. These findings therefore may not be generalizable to urban or more diverse populations. Furthermore, given the lower gestational age and higher proportion of preterm births observed in the overall sample compared with that seen in the population included in the presented analysis, these findings may lack generalizability to gestational weight gain in pregnancies ending in preterm delivery. Additional work is needed in other populations to assess the generalizability of these findings.

## Conclusions

In this cohort study of 1188 individuals, history of weight cycling was associated with both total gestational weight gain and odds of weight gain in excess of IOM guidelines based on prepregnancy BMI. These findings may have important clinical implications for identifying individuals at high risk of excessive gestational weight gain, and there may be benefit for health care professionals to collect information on weight cycling history in addition to prepregnancy weight during initial prenatal visits. Additional work is needed to assess how patterns of weight change during the preconception period are associated with gestational weight gain and the possible short- and long-term impacts on maternal and child health, such as postpartum weight retention and early child growth.

## Notes

Supplement 1. **eFigure 1.** Directed acyclic graph (DAG) used to identify model covariates for the adjusted association between history of weight cycling and gestational weight gain **eFigure 2.** Distribution of (A) self-reported highest BMI in adulthood, excluding pregnancy, (B) self-reported pre-pregnancy BMI for this pregnancy, and (C) gestational weight gain for this pregnancy among 1,188 study participants **eFigure 3.** Sensitivity analysis among participants with available first trimester (<12 weeks) weights abstracted from prenatal medical records **eFigure 4.** Exploratory analysis assessing effect modification by pre-pregnancy weight status on covariate-adjusted associations between history of weight cycling and (A) total gestational weight gain and (B) odds of excessive gestational weight gain (relative to adequate gestational weight gain) per Institute of Medicine guidelines **eFigure 5.** Binned residuals plotted against predicted values for the multinomial logistic regression model of the association between history of weight cycling (predictor, categorical) and i) inadequate versus adequate or ii) excessive versus adequate gestational weight gain relative to adequate gestational weight gain per IOM guidelines for weight gain based on pre-pregnancy BMI categories **eTable 1.** Comparison of subject demographics among the full cohort and participants included in the analysis for this paper **eTable 2.** Distribution of exposure and outcome by missingness **eTable 3.** Sensitivity analyses for the association between history of weight cycling and continuous gestational weight gain **eTable 4.** Comparison of subject demographics among participants with and without available weights abstracted from medical records prior to the second trimester **eTable 5.** Sample size for exploratory analysis stratified by pre-pregnancy weight status **eTable 6.** Model diagnostics for multinomial logistic regression of IOM guidelines for gestational weight gain **eTable 7.** Sensitivity analyses for the association between history of weight cycling and odds of excessive relative to adequate gestational weight gain per IOM guidelines

Supplement 2.
Data Sharing Statement

## References

[1] Rasmussen KM, Catalano PM, Yaktine AL. New guidelines for weight gain during pregnancy: what obstetrician/gynecologists should know. Curr Opin Obstet Gynecol. 2009;21(6):521-526. doi:10.1097/GCO.0b013e328332d24e 19809317 PMC2847829

[2] Langley-Evans SC, Pearce J, Ellis S. Overweight, obesity and excessive weight gain in pregnancy as risk factors for adverse pregnancy outcomes: a narrative review. J Hum Nutr Diet. 2022;35(2):250-264. doi:10.1111/jhn.12999 35239212 PMC9311414

[3] Sebire NJ, Jolly M, Harris JP, . Maternal obesity and pregnancy outcome: a study of 287,213 pregnancies in London. Int J Obes Relat Metab Disord. 2001;25(8):1175-1182. doi:10.1038/sj.ijo.0801670 11477502

[4] Santos S, Voerman E, Amiano P, . Impact of maternal body mass index and gestational weight gain on pregnancy complications: an individual participant data meta-analysis of European, North American and Australian cohorts. BJOG. 2019;126(8):984-995. doi:10.1111/1471-0528.15661 30786138 PMC6554069

[5] Johnson J, Clifton RG, Roberts JM, ; Eunice Kennedy Shriver National Institute of Child Health and Human Development (NICHD) Maternal-Fetal Medicine Units (MFMU) Network. Pregnancy outcomes with weight gain above or below the 2009 Institute of Medicine guidelines. Obstet Gynecol. 2013;121(5):969-975. doi:10.1097/AOG.0b013e31828aea03 23635732 PMC3971915

[6] Melchor I, Burgos J, Del Campo A, Aiartzaguena A, Gutiérrez J, Melchor JC. Effect of maternal obesity on pregnancy outcomes in women delivering singleton babies: a historical cohort study. J Perinat Med. 2019;47(6):625-630. doi:10.1515/jpm-2019-0103 31141492

[7] Djelantik AAAMJ, Kunst AE, van der Wal MF, Smit HA, Vrijkotte TGM. Contribution of overweight and obesity to the occurrence of adverse pregnancy outcomes in a multi-ethnic cohort: population attributive fractions for Amsterdam. BJOG. 2012;119(3):283-290. doi:10.1111/j.1471-0528.2011.03205.x 22168897

[8] Yang Z, Phung H, Freebairn L, Sexton R, Raulli A, Kelly P. Contribution of maternal overweight and obesity to the occurrence of adverse pregnancy outcomes. Aust N Z J Obstet Gynaecol. 2019;59(3):367-374. doi:10.1111/ajo.12866 30024043

[9] Rong K, Yu K, Han X, . Pre-pregnancy BMI, gestational weight gain and postpartum weight retention: a meta-analysis of observational studies. Public Health Nutr. 2015;18(12):2172-2182. doi:10.1017/S1368980014002523 25411780 PMC10271485

[10] Montani JP, Schutz Y, Dulloo AG. Dieting and weight cycling as risk factors for cardiometabolic diseases: who is really at risk? Obes Rev. 2015;16(S1). doi:10.1111/obr.12251 25614199

[11] Sares-Jäske L, Knekt P, Männistö S, Lindfors O, Heliövaara M. Self-report dieting and long-term changes in body mass index and waist circumference. Obes Sci Pract. 2019;5(4):291-303. doi:10.1002/osp4.336 31452914 PMC6700513

[12] Miles-Chan JL, Isacco L. Weight cycling practices in sport: a risk factor for later obesity? Obes Rev. 2021;22(S2)(suppl 2):e13188. doi:10.1111/obr.13188 33372395

[13] Sanaya N, Janusaite M, Dalamaga M, Magkos F. The physiological effects of weight-cycling: a review of current evidence. Curr Obes Rep. 2024;13(1):35-50. doi:10.1007/s13679-023-00539-8 38172475

[14] Field AE, Manson JAE, Laird N, Williamson DF, Willett WC, Colditz GA. Weight cycling and the risk of developing type 2 diabetes among adult women in the United States. Obes Res. 2004;12(2). doi:10.1038/oby.2004.34 14981219

[15] Kim SH, Kwak JS, Kim SP, Choi SH, Yoon HJ. The association between diabetes and hypertension with the number and extent of weight cycles determined from 6 million participants. Sci Rep. 2022;12(1):5235. doi:10.1038/s41598-022-09221-w 35347191 PMC8960790

[16] Zou H, Yin P, Liu L, . Association between weight cycling and risk of developing diabetes in adults: a systematic review and meta-analysis. J Diabetes Investig. 2021;12(4):625-632. doi:10.1111/jdi.13380 32745374 PMC8015818

[17] Bangalore S, Fayyad R, Laskey R, DeMicco DA, Messerli FH, Waters DD. Body-weight fluctuations and outcomes in coronary disease. N Engl J Med. 2017;376(14):1332-1340. doi:10.1056/NEJMoa1606148 28379800

[18] Delahanty LM, Pan Q, Jablonski KA, ; Diabetes Prevention Program Research Group. Effects of weight loss, weight cycling, and weight loss maintenance on diabetes incidence and change in cardiometabolic traits in the Diabetes Prevention Program. Diabetes Care. 2014;37(10):2738-2745. doi:10.2337/dc14-0018 25024396 PMC4170126

[19] Winn NC, Cottam MA, Bhanot M, . Weight cycling impairs pancreatic insulin secretion but does not perturb whole-body insulin action in mice with diet-induced obesity. diabetes. 2022;71(11):2313-2330. doi:10.2337/db22-0161 35802127 PMC9630085

[20] Simonds SE, Pryor JT, Cowley MA. Repeated weight cycling in obese mice causes increased appetite and glucose intolerance. Physiol Behav. 2018;194:184-190. doi:10.1016/j.physbeh.2018.05.026 29842854

[21] Cottam MA, Caslin HL, Winn NC, Hasty AH. Multiomics reveals persistence of obesity-associated immune cell phenotypes in adipose tissue during weight loss and weight regain in mice. Nat Commun. 2022;13(1):2950. doi:10.1038/s41467-022-30646-4 35618862 PMC9135744

[22] Li X, Jiang L, Yang M, Wu YW, Sun JZ. Impact of weight cycling on CTRP3 expression, adipose tissue inflammation and insulin sensitivity in C57BL/6J mice. Exp Ther Med. 2018;16(3):2052-2059. doi:10.3892/etm.2018.6399 30186439 PMC6122336

[23] Gilbert-Diamond D, Cottingham KL, Gruber JF, . Rice consumption contributes to arsenic exposure in US women. Proc Natl Acad Sci U S A. 2011;108(51):20656-20660. doi:10.1073/pnas.1109127108 22143778 PMC3251121

[24] Field AE, Aneja P, Austin SB, Shrier LA, de Moor C, Gordon-Larsen P. Race and gender differences in the association of dieting and gains in BMI among young adults. Obesity (Silver Spring). 2007;15(2):456-464. doi:10.1038/oby.2007.560 17299119

[25] Bannuru RR, ElSayed NA, Aroda VR, . Weight stigma and bias: standards of care in overweight and obesity-2025. BMJ Open Diabetes Res Care. 2025;13(3). doi:10.1136/bmjdrc-2025-004962 40360274

[26] Xiong X, Demianczuk NN, Saunders LD, Wang FL, Fraser WD. Impact of preeclampsia and gestational hypertension on birth weight by gestational age. Am J Epidemiol. 2002;155(3):203-209. doi:10.1093/aje/155.3.203 11821244

[27] Boney CM, Verma A, Tucker R, Vohr BR. Metabolic syndrome in childhood: association with birth weight, maternal obesity, and gestational diabetes mellitus. Pediatrics. 2005;115(3):e290-e296. doi:10.1542/peds.2004-1808 15741354

[28] Gillman MW, Rifas-Shiman S, Berkey CS, Field AE, Colditz GA. Maternal gestational diabetes, birth weight, and adolescent obesity. Pediatrics. 2003;111(3):e221-e226. doi:10.1542/peds.111.3.e221 12612275

[29] Maya J, Pant D, Fu Y, . Gestational weight gain and pregnancy outcomes after GLP-1 receptor agonist discontinuation. JAMA. 2025;334(24):2186-2196. doi:10.1001/jama.2025.20951 41284263 PMC12645404

[30] Lapolla A, Marangon M, Dalfrà MG, . Pregnancy outcome in morbidly obese women before and after laparoscopic gastric banding. Obes Surg. 2010;20(9):1251-1257. doi:10.1007/s11695-010-0199-7 20524157

[31] Gagnon G, Carreau AM, Cloutier-Langevin C, . Trimester-specific gestational weight gain in women with and without previous bariatric surgeries. Eur J Obstet Gynecol Reprod Biol. 2022;270:252-258. doi:10.1016/j.ejogrb.2021.12.033 35000759

[32] Iacovou C, Maric T, Bourke M, Patel D, Savvidou M. Gestational weight gain in pregnancies following bariatric surgery. Obes Surg. 2023;33(4):1004-1011. doi:10.1007/s11695-023-06496-4 36811750 PMC10079746

[33] Headen I, Cohen AK, Mujahid M, Abrams B. The accuracy of self-reported pregnancy-related weight: a systematic review. Obes Rev. 2017;18(3):350-369. doi:10.1111/obr.12486 28170169

[34] Bannon AL, Waring ME, Leung K, . Comparison of self-reported and measured pre-pregnancy weight: implications for gestational weight gain counseling. Matern Child Health J. 2017;21(7):1469-1478. doi:10.1007/s10995-017-2266-3 28155023

