# Supplementary material for: Maternal History of Weight Loss and Prospective Gestational Weight Gain
Source: JAMA Netw Open. 2026 Apr 20;9(4):e267931. doi: 10.1001/jamanetworkopen.2026.7931 (PMC13096973; doi:10.1001/jamanetworkopen.2026.7931)
Supplement: Supplement 1. — eFigure 1. Directed acyclic graph (DAG) used to identify model covariates for the adjusted association between history of weight cycling and gestational weight gain eFigure 2. Distribution of (A) self-reported highest BMI in adulthood, excluding pregnancy, (B) self-reported pre-pregnancy BMI for this pregnancy, and (C) gestational weight gain for this pregnancy among 1,188 study participants eFigure 3. Sensitivity analysis among participants with available first trimester (<12 weeks) weights abstracted from prenatal medical records eFigure 4. Exploratory analysis assessing effect modification by pre-pregnancy weight status on covariate-adjusted associations between history of weight cycling and (A) total gestational weight gain and (B) odds of excessive gestational weight gain (relative to adequate gestational weight gain) per Institute of Medicine guidelines eFigure 5. Binned residuals plotted against predicted values for the multinomial logistic regression model of the association between history of weight cycling (predictor, categorical) and i) inadequate versus adequate or ii) excessive versus adequate gestational weight gain relative to adequate gestational weight gain per IOM guidelines for weight gain based on pre-pregnancy BMI categories eTable 1. Comparison of subject demographics among the full cohort and participants included in the analysis for this paper eTable 2. Distribution of exposure and outcome by missingness eTable 3. Sensitivity analyses for the association between history of weight cycling and continuous gestational weight gain eTable 4. Comparison of subject demographics among participants with and without available weights abstracted from medical records prior to the second trimester eTable 5. Sample size for exploratory analysis stratified by pre-pregnancy weight status eTable 6. Model diagnostics for multinomial logistic regression of IOM guidelines for gestational weight gain eTable 7. Sensitivity analyses for the association between histo [file jamanetwopen-e267931-s001.pdf]

Supplementary Online Content

Muse ME, Gilbert-Diamond D, Madan J, Peacock JL, Karagas MR, Howe CG. Maternal history of weight loss and prospective gestational weight gain. *JAMA Netw Open*. 2026;9(4):267931. doi:10.1001/jamanetworkopen.2026.7931

eFigure 1. Directed acyclic graph (DAG) used to identify model covariates for the adjusted association between history of weight cycling and gestational weight gain
eFigure 2. Distribution of (A) self-reported highest BMI in adulthood, excluding pregnancy, (B) self-reported pre-pregnancy BMI for this pregnancy, and (C) gestational weight gain for this pregnancy among 1,188 study participants
eFigure 3. Sensitivity analysis among participants with available first trimester (<12 weeks) weights abstracted from prenatal medical records
eFigure 4. Exploratory analysis assessing effect modification by pre-pregnancy weight status on covariate-adjusted associations between history of weight cycling and (A) total gestational weight gain and (B) odds of excessive gestational weight gain (relative to adequate gestational weight gain) per Institute of Medicine guidelines
eFigure 5. Binned residuals plotted against predicted values for the multinomial logistic regression model of the association between history of weight cycling (predictor, categorical) and i) inadequate versus adequate or ii) excessive versus adequate gestational weight gain relative to adequate gestational weight gain per IOM guidelines for weight gain based on pre-pregnancy BMI categories
eTable 1. Comparison of subject demographics among the full cohort and participants included in the analysis for this paper
eTable 2. Distribution of exposure and outcome by missingness
eTable 3. Sensitivity analyses for the association between history of weight cycling and continuous gestational weight gain
eTable 4. Comparison of subject demographics among participants with and without available weights abstracted from medical records prior to the second trimester
eTable 5. Sample size for exploratory analysis stratified by pre-pregnancy weight status
eTable 6. Model diagnostics for multinomial logistic regression of IOM guidelines for gestational weight gain
eTable 7. Sensitivity analyses for the association between history of weight cycling and odds of excessive relative to adequate gestational weight gain per IOM guidelines

This supplementary material has been provided by the authors to give readers additional information about their work.

© 2026 Muse ME et al. *JAMA Network Open*.

eFigure 1. Directed acyclic graph (DAG) used to identify model covariates for the adjusted association between history of weight cycling and gestational weight gain. Factors shown in white reflect covariates included in final models. Lines in green reflect the hypothesized causal path between weight cycling and gestational weight gain.

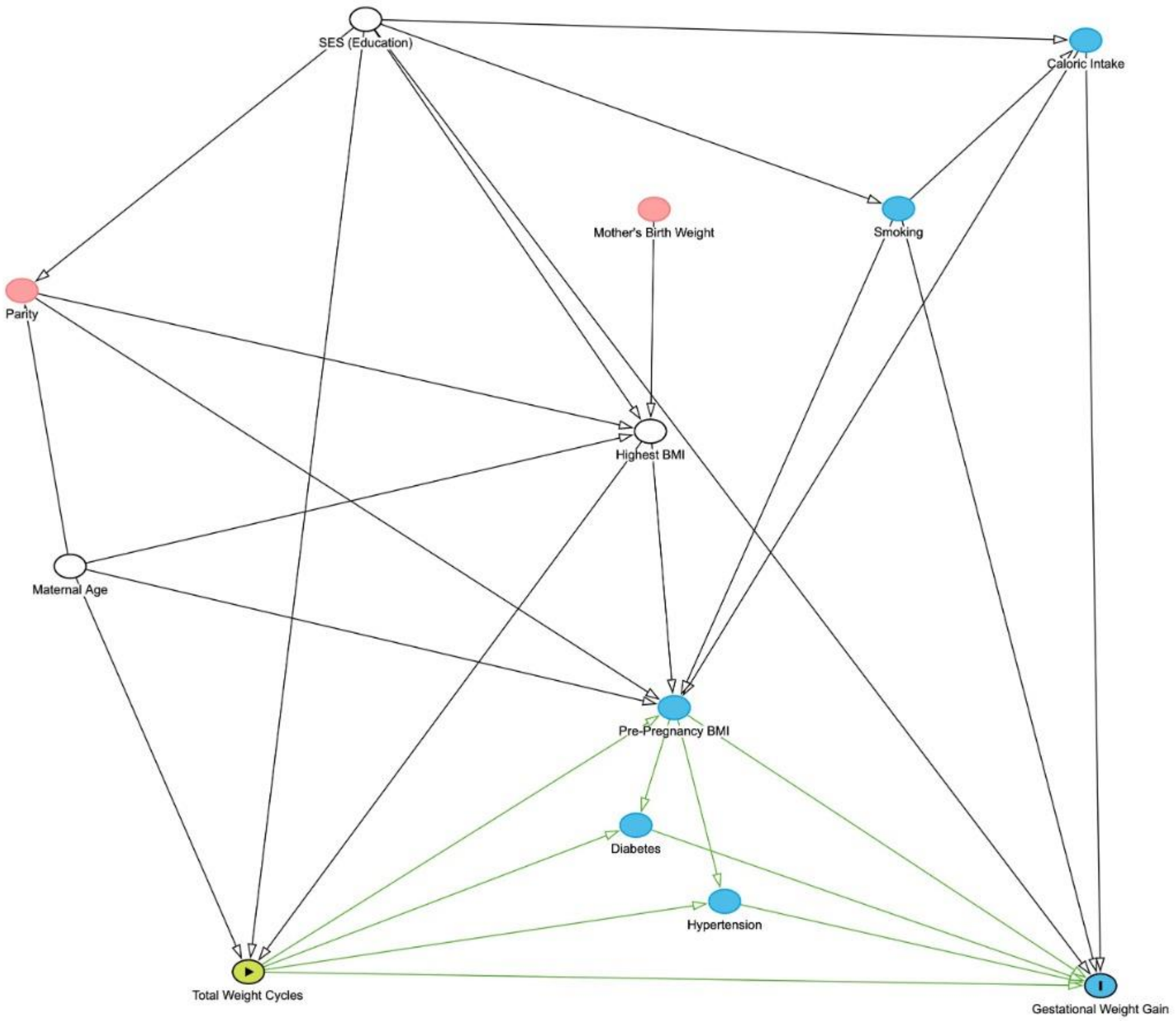

© 2026 Muse ME et al. *JAMA Network Open*.

eFigure 2. Distribution of (A) self-reported highest BMI in adulthood, excluding pregnancy, (B) self-reported pre-pregnancy BMI for this pregnancy, and (C) gestational weight gain for this pregnancy among 1,188 study participants.

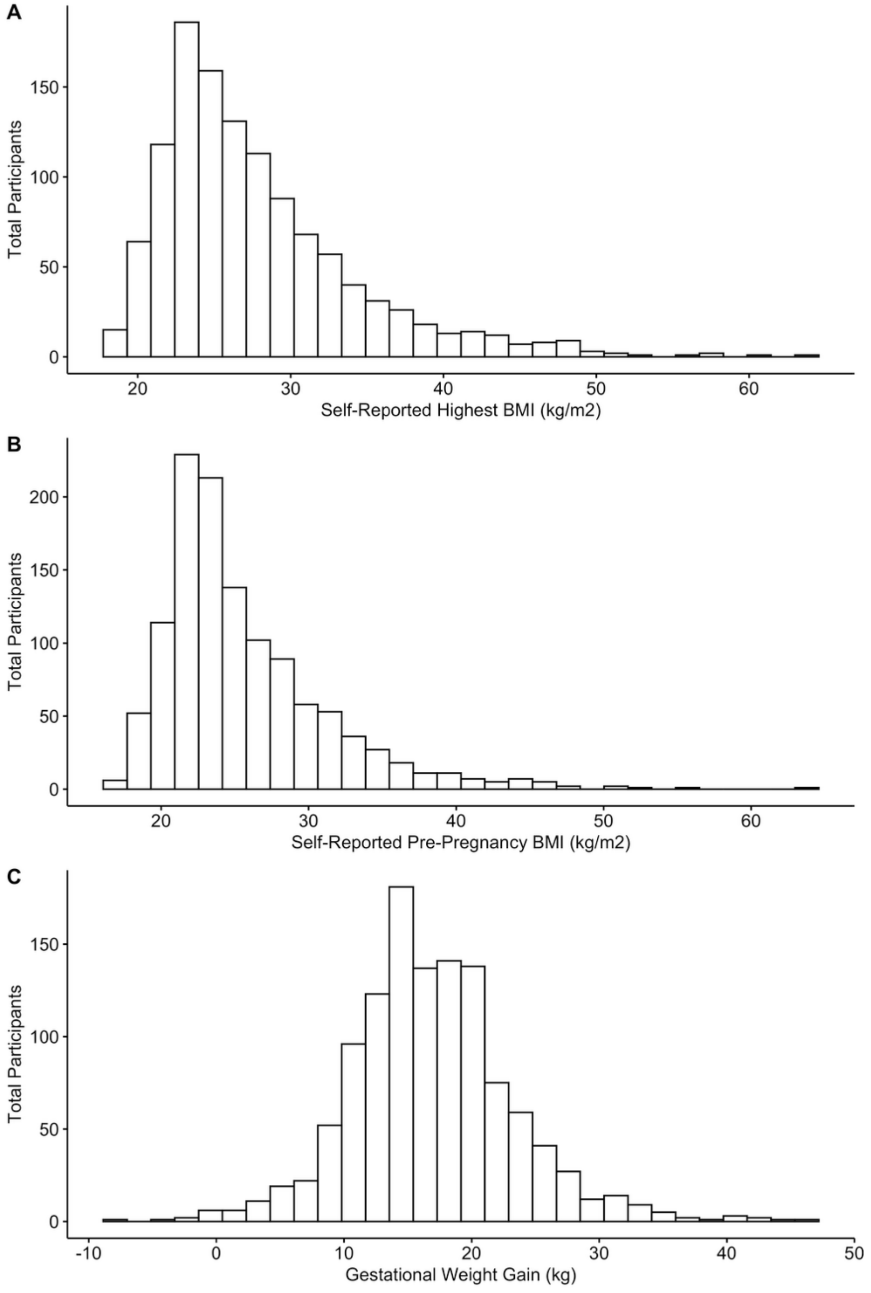

© 2026 Muse ME et al. *JAMA Network Open*.

eFigure 3. Sensitivity analysis among participants with available first trimester (<12 weeks) weights abstracted from prenatal medical records (PNMR; n = 1,078). (A) Distribution of gestational age at first pregnancy weight abstracted from PNMR by weight cycling history. (B) Distribution of the difference between first pregnancy weight abstracted from PNMR and self-reported pre-pregnancy weight by weight cycling history. Covariate-adjusted associations between history of weight cycling and total gestational weight gain calculated using (C) self-reported pre-pregnancy weight and (D) first trimester weight abstracted from PNMR. Covariate-adjusted associations between history of weight cycling and odds of excessive gestational weight gain (relative to adequate gestational weight gain) per Institute of Medicine guidelines calculated using (E) self-reported pre-pregnancy weight and (F) first trimester weight abstracted from PNMR. Models are adjusted for self-reported highest attained BMI in adulthood (excluding pregnancy; continuous; modeled with restricted cubic splines with 4 knots), educational attainment (categorical: high school graduate or less, some college, and college graduate or more), smoking history (categorical: ever, never, not reported), and maternal age (continuous). P_{trend} reflects the P value for the linear term when history of weight cycling is treated as an ordinal variable.

© 2026 Muse ME et al. *JAMA Network Open*.

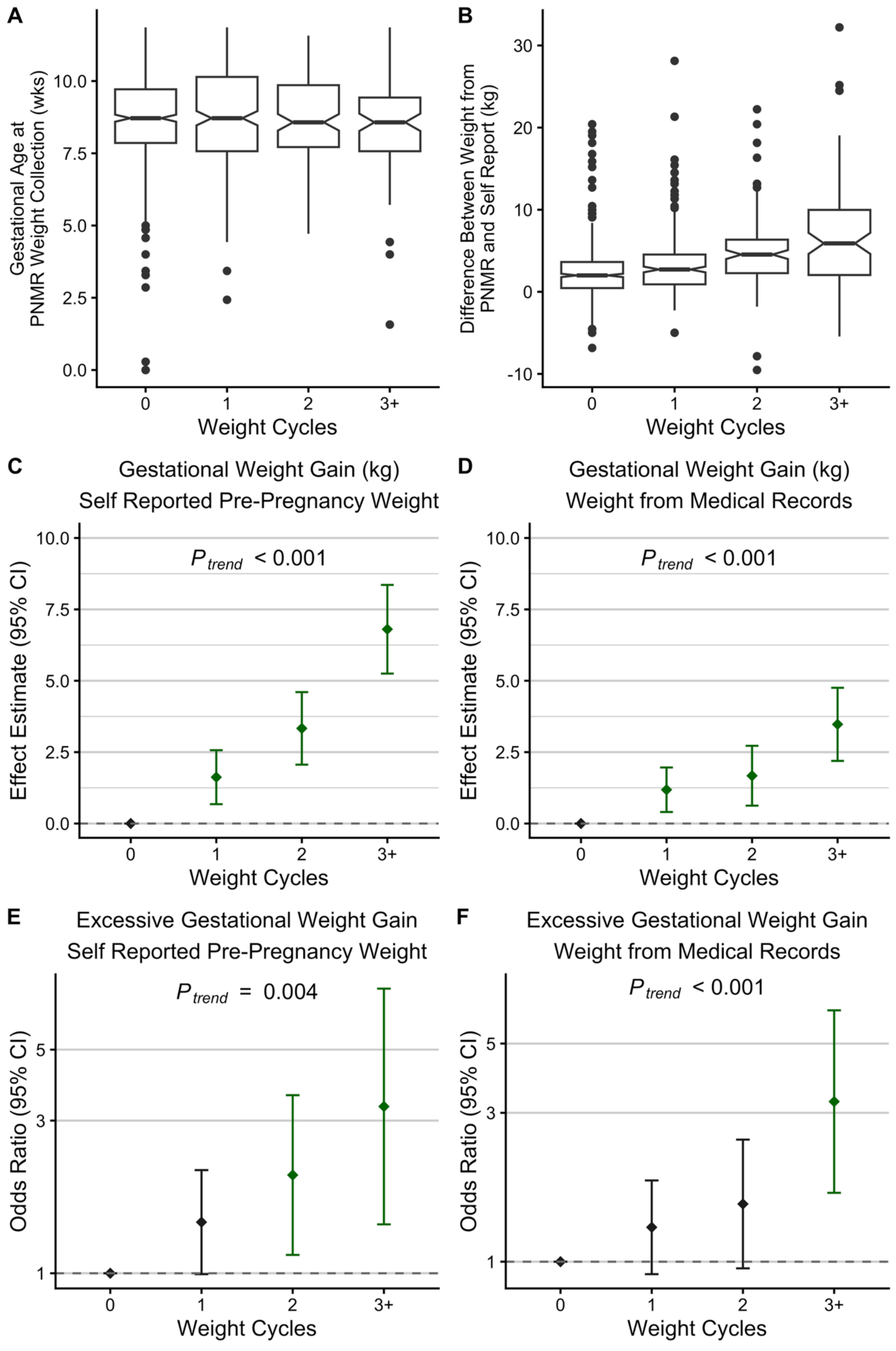

© 2026 Muse ME et al. *JAMA Network Open*.

eFigure 4. Exploratory analysis assessing effect modification by pre-pregnancy weight status on covariate-adjusted associations between history of weight cycling and (A) total gestational weight gain and (B) odds of excessive gestational weight gain (relative to adequate gestational weight gain) per Institute of Medicine guidelines. Models are adjusted for educational attainment (categorical: high school graduate or less, some college, and college graduate or more), and maternal age (continuous). Overall models additionally adjust for pre-pregnancy weight status (binary, 18.5 < BMI < 25 kg/m^2, BMI ≥ 25 kg/m^2). P_{int} reflects the P value from a likelihood ratio test comparing the overall model to a nested model with an interaction term between weight cycling history and pre-pregnancy weight status.

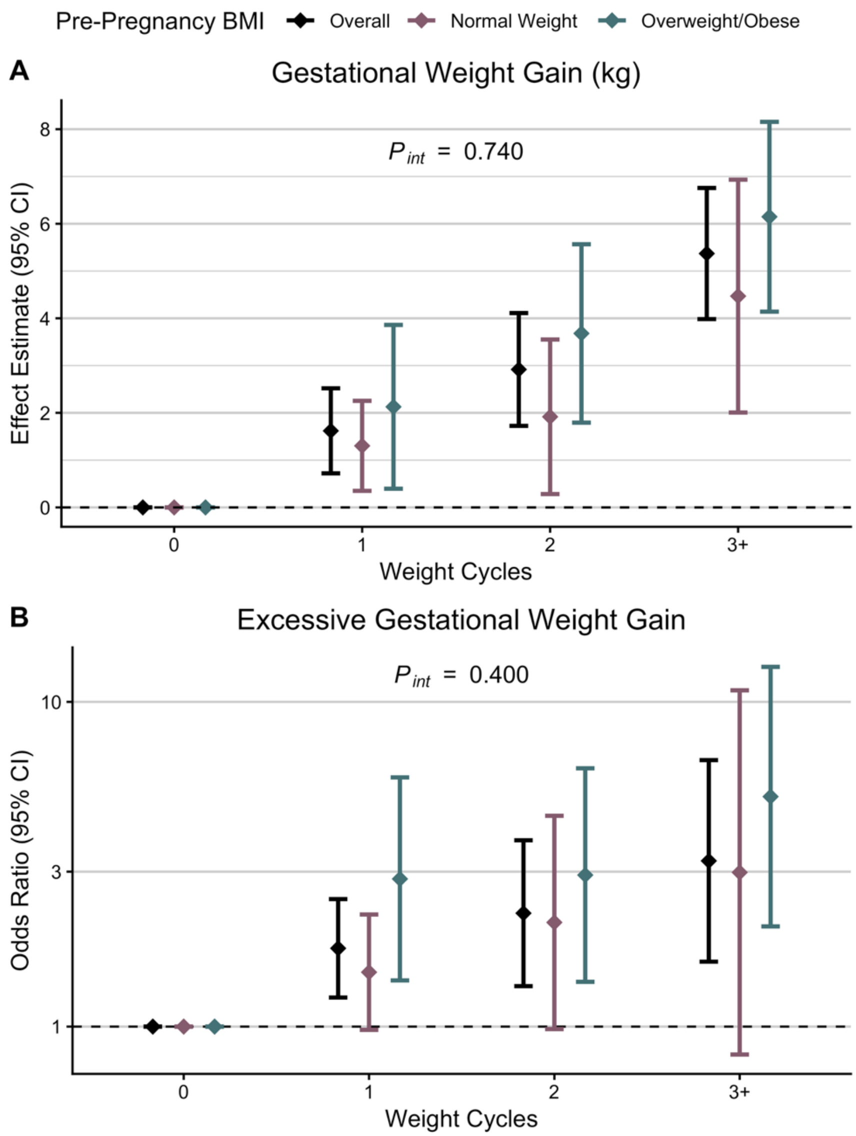

© 2026 Muse ME et al. *JAMA Network Open*.

eFigure 5. Binned residuals plotted against predicted values for the multinomial logistic regression model of the association between history of weight cycling (predictor, categorical) and i) inadequate versus adequate or ii) excessive versus adequate gestational weight gain relative to adequate gestational weight gain per IOM guidelines for weight gain based on pre-pregnancy BMI categories. Models are additionally adjusted for self-reported highest attained BMI in adulthood (excluding pregnancy; continuous; modeled with restricted cubic splines with 4 knots), educational attainment (categorical: high school graduate or less, some college, and college graduate or more), smoking history (categorical: ever, never, not reported), and maternal age (continuous).

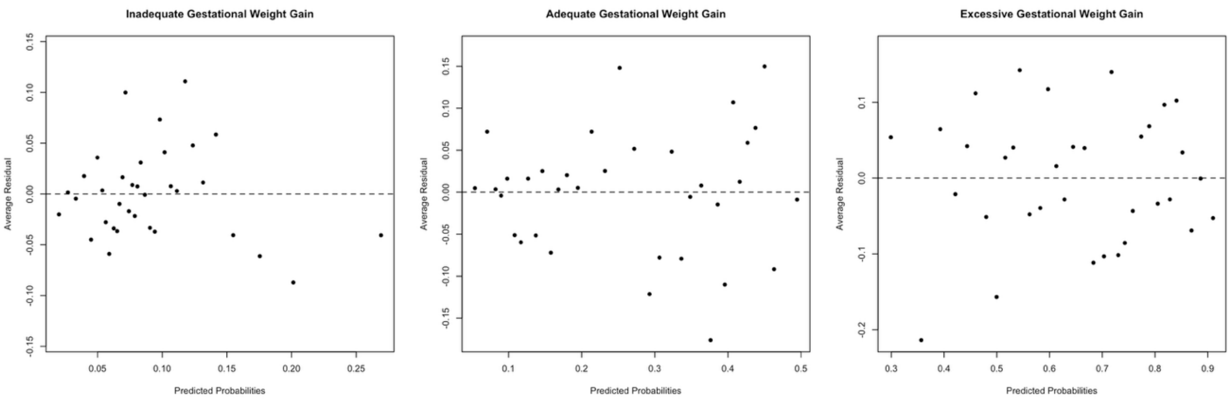

© 2026 Muse ME et al. *JAMA Network Open*.

eTable 1. Comparison of subject demographics among the full cohort and participants included in the analysis for this paper

	Excluded Participants[1]	Analytic Subset	P Value	Excluded Participant[1] Missingness	Analytic Subset Missingness
**Sample Size**	1063	1188			
**Age** mean (SD)	31.11 (4.98)	31.49 (4.67)	0.058	2	0
**Educational Attainment** n (%)			0.031	411	0
High School Graduate or Less	101 (15.5)	135 (11.4)			
Some College	110 (16.9)	194 (16.3)			
College Graduate or More	441 (67.6)	859 (72.3)			
**Race** n (%)			0.069	239	2
American Indian or Alaska Native	4 (0.5)	5 (0.4)			
Asian	14 (1.7)	8 (0.7)			
Black	2 (0.2)	2 (0.2)			
White	797 (96.7)	1148 (96.8)			
Mixed Race	7 (0.8)	23 (1.9)			
**Ethnicity** n (%)			<0.001	257	9
Hispanic	17 (2.1)	27 (2.3)			
Non-Hispanic	762 (86.8)	1118 (94.1)			
**Parity** n (%)			0.363	210	3
1	368 (43.1)	518 (43.7)			
2	305 (35.8)	446 (37.6)			
3+	180 (21.1)	221 (18.6)			
**Smoking Status** n (%)			<0.001	585	106
Never Smoker	389 (81.4)	963 (89.0)			
Ever Smoker	89 (18.6)	119 (11.0)			
**Highest Adult BMI, kg/m^2** mean (SD)	27.53 (7.45)	27.79 (6.48)	0.462	514	0
**Total Weight Cycles** n (%)			0.839	468	0
0	306 (51.4)	627 (52.8)			
1	158 (26.6)	298 (25.1)			
2	72 (12.1)	153 (12.9)			
3+	59 (9.9)	110 (9.3)			
**Pre-Pregnancy BMI** mean (SD)	25.55 (5.80)	25.58 (5.60)	0.917	510	0
< 18.5 kg/m^2	23 (4.2)	24 (2.0)	0.07		
18.5 – 25 kg/m^2	297 (53.6)	671 (56.5)			
25 – 30 kg/m^2	133 (24.0)	286 (24.1)			
> 30 kg/m^2	101 (18.2)	207 (17.4)			
**Total Weeks Gestation** mean (SD)	38.48 (3.54)	39.15 (1.50)	<0.001	185	0
Premature	116 (13.2)	70 (5.9)	<0.001		
Term	762 (86.8)	1118 (94.1)			
**Total Weight Gain (kg)** mean (SD)	16.69 (9.81)	16.92 (6.47)	0.787	998	0
**Weight Gain by IOM Guidelines** n (%)			0.665	999	0
Inadequate	5 (7.8)	109 (9.2)			
Adequate	20 (31.2)	312 (26.3)			
Excessive	39 (60.9)	767 (64.6)			

[1] Excludes participants who withdrew from the study

© 2026 Muse ME et al. *JAMA Network Open*.

eTable 2. Distribution of exposure and outcome by missingness

Exposure: Weight Cycling	Total (n)	Outcome: Gestational Weight Gain (kg)		
		Missing n (%)	Available n (%)	Mean (sd)
Missing	468	435 (92.9)	33 (7.1)	17.42 (6.97)
Reported	1783	563 (31.6)	1220 (68.4)	16.89 (6.67)
0	933	289 (31.0)	644 (69.0)	16.03 (5.83)
1	456	154 (33.8)	302 (66.2)	17.21 (6.17)
2	225	70 (31.1)	155 (68.9)	18.00 (7.15)
3+	169	50 (29.6)	119 (70.4)	19.34 (9.91)

© 2026 Muse ME et al. *JAMA Network Open*.

eTable 3. Sensitivity analyses for the association between history of weight cycling and continuous gestational weight gain

		Weight Cycles[1]				
		0	1	2	3+	
Sensitivity Analysis[2]	Sample Size	ES[3] (95% CI)	ES[3] (95% CI)	ES[3] (95% CI)	ES[3] (95% CI)	P_{trend}
Excluding Underweight Mothers (n = 24)	1164	Ref.	1.7 (0.7, 2.6)	3.1 (1.9, 4.4)	6.2 (4.7, 7.6)	< 0.001
Excluding Prior Diabetes (n = 45)	1143	Ref.	1.6 (0.7, 2.6)	3.1 (1.9, 4.4)	6.3 (4.8, 7.8)	< 0.001
Excluding Prior Hypertension (n = 218)	970	Ref.	1.2 (0.2, 2.2)	3.1 (1.8, 4.4)	6.2 (4.6, 7.7)	< 0.001
Adjusting for Pre-Pregnancy BMI[4]	1188	Ref.	1.8 (1.0, 2.7)	3.3 (2.1, 4.4)	6.2 (4.8, 7.6)	< 0.001
Without Adjustment for BMI[5]	1188	Ref.	1.2 (0.3, 2.0)	2.1 (1.0, 3.2)	4.2 (2.9, 5.5)	< 0.001

[1] Weight cycles are modeled categorically and are defined as the total number of times a participant reported losing 20 or more pounds excluding following pregnancy
[2] Linear models are adjusted for self-reported highest attained BMI in adulthood (excluding pregnancy; continuous; modeled using restricted cubic splines with 4 knots), educational attainment (categorical: high school graduate or less, some college, and college graduate or more), smoking history (categorical; never, ever, not reported), and maternal age (continuous).
[3] Effect size (ES) units are in kg
[4] Model is adjusted for self-reported pre-pregnancy BMI (kg/m^2; continuous; modeled using restricted cubic splines with 4 knots) instead of self-reported highest attained BMI in adulthood.
[5] Model is adjusted for educational attainment (categorical: high school graduate or less, some college, and college graduate or more), smoking history (categorical; never, ever, not reported), and maternal age (continuous).

© 2026 Muse ME et al. *JAMA Network Open*.

eTable 4. Comparison of subject demographics among participants with and without available weights abstracted from medical records prior to the second trimester

	First Trimester Weight Available		
	Yes	No	P Value
**Sample Size**	1078	110	
**Age** mean (SD)	31.58 (4.60)	30.60 (5.26)	0.035
**Educational Attainment** n (%)			<0.001
High School Graduate or Less	113 (10.5)	22 (20.0)	
Some College	164 (15.2)	30 (27.3)	
College Graduate or More	801 (74.3)	58 (52.7)	
**Race**[2] n (%)			0.497
American Indian or Alaska Native	5 (0.5)	0 (0.0)	
Asian	8 (0.7)	0 (0.0)	
Black	2 (0.2)	0 (0.0)	
White	1043 (96.8)	105 (96.3)	
Mixed Race	19 (1.8)	4 (3.7)	
**Ethnicity**[3] n (%)			0.18
Hispanic	27 (2.5)	0 (0.0)	
Non-Hispanic	1043 (97.5)	109 (100.0)	
**Parity** n (%)			0.002
1	478 (44.5)	40 (36.4)	
2	410 (38.1)	36 (32.7)	
3+	187 (17.4)	34 (30.9)	
**Smoking Status**[4] n (%)			0.275
Never Smoker	876 (89.4)	87 (85.3)	
Ever Smoker	104 (10.6)	15 (14.7)	
**Highest Adult BMI, kg/m^2** mean (SD)	27.67 (6.33)	28.89 (7.76)	0.06
**Total Weight Cycles** n (%)			0.315
0	575 (53.3)	52 (47.3)	
1	268 (24.9)	30 (27.3)	
2	140 (13.0)	13 (11.8)	
3+	95 (8.8)	15 (13.6)	
**Pre-Pregnancy BMI** mean (SD)	25.53 (5.51)	26.10 (6.43)	0.302
< 18.5 kg/m^2	22 (2.0)	2 (1.8)	0.788
18.5 to < 25 kg/m^2	612 (56.8)	59 (53.6)	
25 to < 30 kg/m^2	260 (24.1)	26 (23.6)	
> 30 kg/m^2	184 (17.1)	23 (20.9)	
**Total Weeks Gestation** mean (SD)	39.16 (1.48)	39.09 (1.69)	0.681
**Total Weight Gain (kg)** mean (SD)	16.78 (6.42)	18.26 (6.79)	0.022
**Weight Gain by IOM Guidelines** n (%)			
Inadequate	105 (9.7)	4 (3.6)	0.06
Adequate	286 (26.5)	26 (23.6)	
Excessive	687 (63.7)	80 (72.7)	

[1] Missing for 3 participants
[2] Missing for 2 participants
[3] Missing for 9 participants
[4] Missing for 106 participants

© 2026 Muse ME et al. *JAMA Network Open*.

eTable 5. Sample size for exploratory analysis stratified by pre-pregnancy weight status

	Weight Cycles	Overall<br>BMI > 18.5 kg/m^2	Normal Weight<br>BMI 18.5 - 25 kg/m^2	Overweight or Obese<br>BMI 25+ kg/m^2
All Participants	0	610	457	153
	1	293	152	141
	2	151	44	107
	3+	110	18	92
Adequate Gestational Weight Gain	0	210	178	32
	1	60	47	13
	2	22	10	12
	3+	10	3	7
Excessive Gestational Weight Gain	0	335	226	109
	1	207	91	116
	2	123	32	91
	3+	91	13	78

eTable 6. Model diagnostics for multinomial logistic regression of IOM guidelines for gestational weight gain

		Predicted[1]		
		Inadequate	Adequate	Excessive
Observed[2]	Inadequate	0	26	83
	Adequate	1	69	242
	Excessive	0	49	718

AIC: 1905.2
McFadden Pseudo R^2: 0.08

[1] Models are adjusted for total times total number of times a mother reported losing 20 or more pounds excluding following a pregnancy (predictor; categorical); self-reported highest attained BMI in adulthood (excluding pregnancy; continuous; modeled with restricted cubic splines with 4 knots), educational attainment (categorical: high school graduate or less, some college, and college graduate or more), smoking history (categorical: ever, never, not reported), and maternal age (continuous).
[2] Gestational weight gain relative to Institute of Medicine 2009 guidelines for suggested gestational weight gain based on pre-pregnancy BMI

© 2026 Muse ME et al. *JAMA Network Open*.

eTable 7. Sensitivity analyses for the association between history of weight cycling and odds of excessive relative to adequate gestational weight gain per IOM guidelines

		Weight Cycles[1]				
	Sample	0	1	2	3+	
Sensitivity Analysis[2]	Size	OR (95% CI)	OR (95% CI)	OR (95% CI)	OR (95% CI)	P_{trend}
Excluding Underweight Mothers (n = 24)	1164	Ref.	1.4 (1.0, 2.1)	1.8 (1.0, 3.1)	2.8 (1.3, 6.0)	0.007
Excluding Prior Diabetes (n = 45)	1143	Ref.	1.4 (1.0, 2.0)	1.7 (1.0, 3.0)	2.9 (1.3, 6.3)	0.008
Excluding Prior Hypertension (n = 218)	970	Ref.	1.1 (0.7, 1.6)	1.5 (0.8, 2.7)	2.1 (0.9, 4.8)	0.051
Adjusting for Pre-Pregnancy BMI[3]	1188	Ref.	1.7 (1.2, 2.5)	2.3 (1.4, 3.9)	3.2 (1.6, 6.8)	0.001
Without Adjustment for BMI[4]	1188	Ref.	2.1 (1.5, 2.9)	3.7 (2.3, 6.0)	6.2 (3.1, 12.3)	< 0.001

[1] Weight cycles are modeled categorically and are defined as the total number of times a participant reported losing 20 or more pounds excluding following pregnancy
[2] Multinomial logistic regression models are adjusted for self-reported highest attained BMI in adulthood (excluding pregnancy; continuous; modeled using restricted cubic splines with 4 knots), educational attainment (categorical: high school graduate or less, some college, and college graduate or more), smoking history (categorical; never, ever, not reported), and maternal age (continuous).
[3] Model is adjusted for self-reported pre-pregnancy BMI (kg/m2; continuous; modeled using restricted cubic splines with 4 knots) instead of self-reported highest attained BMI in adulthood.
[4] Model is adjusted for educational attainment (categorical: high school graduate or less, some college, and college graduate or more), smoking history (categorical; never, ever, not reported), and maternal age (continuous).

© 2026 Muse ME et al. *JAMA Network Open*.
