# Supplementary material for: Maternal History of Weight Loss and Prospective Gestational Weight Gain
Source: JAMA Netw Open. 2026 Apr 20;9(4):e267931. doi: 10.1001/jamanetworkopen.2026.7931 (PMC13096973; doi:10.1001/jamanetworkopen.2026.7931)
Supplement: Supplement 2. — Data Sharing Statement [file jamanetwopen-e267931-s002.pdf]

Data Sharing Statement

Muse. Maternal History of Weight Loss and Prospective Gestational Weight Gain. *JAMA Netw Open*. Published April 20, 2026. doi:10.1001/jamanetworkopen.2026.7931

Data

Data available: Data from this study can be made available with the permission of the principal investigator, Dr Margaret Karagas.
